# COVID-19-Associated Neurological Disorders: The Potential Route of CNS Invasion and Blood-Brain Barrier Relevance

**DOI:** 10.3390/cells9112360

**Published:** 2020-10-27

**Authors:** Aneesha Achar, Chaitali Ghosh

**Affiliations:** 1 Cerebrovascular Research, Department of Biomedical Engineering, Lerner Research Institute, Cleveland Clinic, Cleveland, OH 44195, USA; ava28@case.edu; 2 Department of Biomedical Engineering and Molecular Medicine, Cleveland Clinic Lerner College of Medicine of Case Western Reserve University, Cleveland, OH 44195, USA

**Keywords:** CNS, COVID-19, SARS-CoV-2, blood-brain barrier, cerebrovascular, neurological disorders

## Abstract

Severe acute respiratory syndrome coronavirus 2 (SARS-CoV-2) is a novel human coronavirus that has sparked a global pandemic of the coronavirus disease of 2019 (COVID-19). The virus invades human cells through the angiotensin-converting enzyme 2 (ACE2) receptor-driven pathway, primarily targeting the human respiratory tract. However, emerging reports of neurological manifestations demonstrate the neuroinvasive potential of SARS-CoV-2. This review highlights the possible routes by which SARS-CoV-2 may invade the central nervous system (CNS) and provides insight into recent case reports of COVID-19-associated neurological disorders, namely ischaemic stroke, encephalitis, encephalopathy, epilepsy, neurodegenerative diseases, and inflammatory-mediated neurological disorders. We hypothesize that SARS-CoV-2 neuroinvasion, neuroinflammation, and blood-brain barrier (BBB) dysfunction may be implicated in the development of the observed disorders; however, further research is critical to understand the detailed mechanisms and pathway of infectivity behind CNS pathogenesis.

## 1. Introduction

The coronavirus disease of 2019 (COVID-19) is an infectious disease caused by severe acute respiratory syndrome coronavirus 2 (SARS-CoV-2). The first cases of COVID-19 were observed in December 2019 in Wuhan City, Hubei Province of China [1]. Since then, SARS-CoV-2 rapidly spread to over 200 countries and territories [2], and the World Health Organization declared COVID-19 a global pandemic [3]. As of September 4, 2020, approximately 26 million cases of COVID-19 have been reported, resulting in ∼860,000 deaths worldwide [4].

SARS-CoV-2 is a human coronavirus: an enveloped, positive-sensed, ssRNA virus characterized by its crown-like morphology [5]. Other human coronaviruses include HCoV-22E, HCoV-OC43, HCoV-NL63, HCoV-HKU1, severe acute respiratory syndrome coronavirus (SARS-CoV), and Middle East respiratory syndrome coronavirus (MERS-CoV), all of which infect the human respiratory system [6]. Although the majority of human coronaviruses cause only mild symptoms, SARS-CoV, MERS-CoV, and SARS-CoV-2 may be highly fatal [5]. Moreover, while SARS-CoV and MERS-CoV primarily cause infections of the lower respiratory tract [7,8], the novel SARS-CoV-2 causes both upper and lower respiratory tract infections [9].

Symptoms of COVID-19 range from mild to severe conditions. Mild symptoms of COVID-19 include fever, chills, cough, shortness of breath, fatigue, body aches, headache, loss of taste or smell, sore throat, congestion, nausea, and/or vomiting within two weeks of exposure to the virus [10]. Severe illnesses from COVID-19 include pneumonia, acute respiratory distress syndrome and sepsis [11]. Individuals more susceptible to severe illness include those over the age of 65 or currently living in a long-term care facility. However, individuals of all ages are at a higher risk for severe illness if they have underlying medical conditions or suffer from comorbidities such as chronic lung, kidney, or liver disease, moderate to severe asthma, severe heart conditions, diabetes, severe obesity, and conditions that cause immunodeficiency [12].

It has been well-established that SARS-CoV-2 invades human cells through interaction with the human angiotensin-converting enzyme 2 (ACE2) receptor [13]. This mechanism is detailed in the section “Mechanism of Viral Invasion: ACE2 Receptor”. As COVID-19 is primarily a respiratory disease, high levels of ACE2 have been observed in pulmonary type II alveolar cells and respiratory epithelial cells. However, ACE2 expression is not limited to the respiratory tract. Other areas that have been found to exhibit high ACE2 expression include myocardial cells, ileal and esophageal epithelial cells, renal proximal tubule cells, and bladder urothelial cells. Therefore, the ACE2 receptors function and overactivity may influence such target cells and organs, increasing the vulnerability to infection [14].

More recently, reports have emerged of the neuroinvasive potential of SARS-CoV-2 [15,16,17,18,19]. In a retrospective study performed on COVID-19 patients from Wuhan, China, neurological symptoms were observed in 36.4% of total patients and 45.5% of patients with severe infections [15]. Observed symptoms of central nervous system (CNS) infection included dizziness, headache, impaired consciousness, acute cerebrovascular disease, and ataxia [15,18]. Additionally, a large-scale study of COVID-19 patients from the United Kingdom revealed neurological disorders were frequent among the 153 “unique” cases of COVID-19 identified by physicians. Of the 125 patients with clinical data, a cerebrovascular event, defined as ischaemic stroke, intracerebral haemorrhage, or CNS vasculitis, was observed in 62% of patients reported. An altered mental status, defined as unspecified encephalopathy, encephalitis, or psychiatric diagnosis, was observed in 31% of patients [16]. Moreover, the presentations of COVID-19-associated neurological disorders were observed to fit into five distinct categories: encephalopathies, inflammatory CNS syndromes, ischaemic strokes, peripheral neurological disorders, and miscellaneous other CNS disorders [17]. As neurological manifestations have become increasingly prevalent in recent times, evidence demonstrates that illness from COVID-19 may go beyond that of respiratory tract infectivity.

Within this review, we highlight the possible pathways by which SARS-CoV-2 may invade the brain and briefly discuss the recent case reports of neurological disorders associated with COVID-19 illness, focusing primarily on CNS manifestations.

## 2. Gateway to the Brain: The Blood-Brain Barrier as a Possible Entry Route

There are two primary pathways by which SARS-CoV-2 may reach the CNS. First, the virus may travel by retrograde axonal transport via the cribriform plate. In this pathway, SARS-CoV-2 present in the nasal endothelium may adhere to motor proteins along sensory and olfactory nerves to the brain [20]. Evidence of this pathway includes loss of smell [21]. An observational study of 114 COVID-19 patients from the *Nord Franche-Comté* hospital revealed that anosmia was observed among 47% of patients [22]. The incidence of olfactory dysfunction varies greatly, ranging from 33.9% to 68% between countries [23]. Moreover, analysis by Brann et al. revealed the expression of ACE2 in human olfactory epithelium sustentacular cells, horizontal basal cells, and Bowman’s gland, providing a potential explanation for COVID-19-associated anosmia and further evidence of this pathway [24].

An alternative route, as a second path to the CNS is through the dissemination of SARS-CoV-2 into the systemic circulatory system following infection of the respiratory tract [25]. In this pathway, SARS-CoV-2 may spread to other target tissues and organs with the cerebral blood flow. However, the virus cannot simply migrate from the capillaries of the systemic circulatory system to the brain through endothelial cells due to the unique physiology of the blood-brain barrier (BBB). The BBB is a semi-permeable barrier between the blood vessels and brain parenchyma that vascularizes the CNS and strictly regulates which molecules may pass to the brain. The blood vessels consist primarily of endothelial cells, which form the unique tight junctions to prevent the leakage of molecules and ions into the brain [26,27,28,29]. Surrounding the brain endothelial walls are astrocytes, pericytes, and, a few microns away, the neuronal termini. Pericytes, along with endothelial cells, are responsible for controlling the invasion of immune cells. Astrocytes, a type of glial cell, extend long cellular processes that surround the blood vessel. Astrocytes also serve as the vital link between the cardiovascular and nervous systems, regulating blood flow and the activity of vascular smooth muscle cells. The function of the BBB is also affected by immune cells, namely perivascular macrophages, leukocytes and microglial cells. These cells are involved in the innate immune response to pathogens [28]. Evidently, the BBB is a physiologically and metabolically active interface forming the neurovascular unit [27,30,31,32].

**Figure 1:**
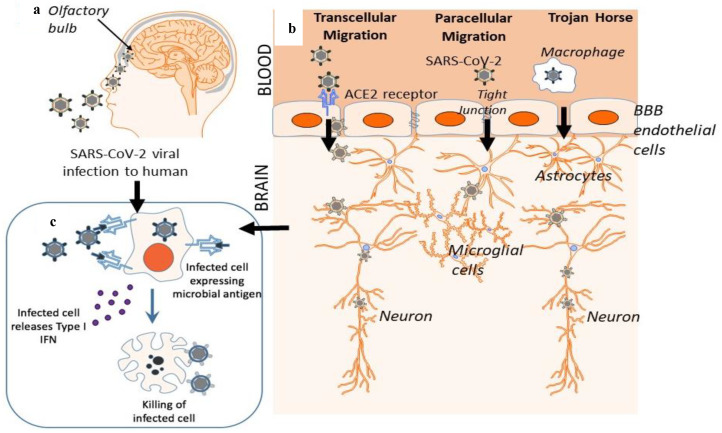
Potential routes of severe acute respiratory syndrome coronavirus 2 (SARS-CoV-2) to the central nervous system (CNS) and preliminary activation of the immune system. (**a**) Once SARS-CoV-2 is inhaled into the nasal cavity, the virus may travel to the CNS by retrograde axonal transport along sensory and olfactory nerves via the cribriform plate, a bone structure located nearby the olfactory bulb. In this pathway, SARS-CoV-2 would bypass the blood-brain barrier (BBB). (**b**) Following a respiratory tract infection characteristic of the virus, SARS-CoV-2 may disseminate into the systemic circulatory system. Upon reaching the BBB, SARS-CoV-2 may invade host endothelial cells by interaction with the angiotensin-converting enzyme 2 (ACE2) receptor, altering tight junction proteins formed by BBB endothelial cells, or phagocytosis by immune cells. These three mechanisms are termed transcellular migration, paracellular migration, and the Trojan horse strategy, respectively. (**c**) In both pathways, cells infected with SARS-CoV-2 release type I interferons, which alert neighboring and immune cells to the presence of pathogen. Under normal conditions, infected cells are eliminated by host immune cells to prevent further replication and the spread of SARS-CoV-2.

There are three main mechanisms by which a virus may cross the BBB: transcellular migration, paracellular migration, and the “Trojan horse” strategy [33]. During transcellular migration, viruses invade host endothelial cells to cross the BBB. During paracellular migration, viruses invade tight junctions formed by endothelial cells of the BBB. [34]. During the Trojan horse strategy, a virus is engulfed by phagocytic host cells, such as neutrophils and macrophages. SARS-CoV-2 may utilize either a single or combination of these mechanisms [33], which must be further investigated. Figure 1 schematically depicts the two potential routes of CNS invasion: axonal transport through the olfactory epithelium or dissemination into the systemic circulatory system and subsequent crossing of the BBB. Both pathways ultimately result in activation of the immune system. The ACE2 receptor and regulation process is therefore a critical entry route that may be associated with the propagation of infection and disease pathogenesis.

## 3. Mechanism of Cellular Viral Invasion: ACE2 Receptor

While SARS-CoV-2 is a novel virus, the cell entry mechanism of SARS-CoV, the predecessor to SARS-CoV-2, is well-established. The receptor binding domain of SARS-CoV binds to the human ACE2 receptor on the cell surface [13]. The receptor binding domain is found on the S1 subunit of the homotrimeric spike protein expressed by the virus [35]. After the interaction between the receptor binding domain and ACE2, the spike protein is proteolytically cleaved along its S1/S2 boundary by transmembrane serine protease 2 and lysosomal protease cathepsins [36]. The S1 subunit dissociates with ACE2. The S2 subunit increases in stability, a step critical for the fusion of the viral and cell membrane [13].

Like SARS-CoV, the novel coronavirus SARS-CoV-2 also invades cells through the ACE2 receptor [37], suggesting a similar mechanism of entry. However, while the receptor binding domain of SARS-CoV-2 has a higher binding affinity for ACE2 than that of SARS-CoV, it is less accessible. This is because the receptor binding domain of the novel coronavirus exists primarily in its “lying” conformation, compared to the “standing” conformation characteristic of SARS-CoV [36]. Additionally, SARS-CoV-2 also uses transmembrane serine protease 2 for S protein cleavage and priming [38]. However, SARS-CoV-2 entry is also facilitated by furin preactivation, allowing SARS-CoV-2 to enter cells with low levels of transmembrane serine protease 2 and lysosomal protease cathepsin [36].

Once SARS-CoV-2 has gained entry into the cell, the virus begins replication in the cytoplasm. First, an RNA-dependent RNA polymerase specific to SARS-CoV-2 transcribes the genome. The resulting antigenome template is further transcribed to produce positive-strand RNA and mRNAs, which produce peptides after capping and polyadenylation. Viral RNA is spread to neighboring cells through the exocytosis of virions [39].

## 4. Cytokine Storm on the Blood-Brain Barrier: A Malfunction of the Immune System

Cells infected with SARS-CoV-2 may produce type I interferons, cytokines that alert the immune system to the presence of pathogen and induce an antimicrobial state in neighboring cells [40]. Host cells along the BBB also have pattern recognition receptors with the ability to recognize pathogen-associated molecular patterns within the systemic circulatory system [33]. For example, Toll-like receptors 7 and 8 recognize ssRNA, a pathogen-associated molecular pattern associated with SARS-CoV-2 [41].

Recognition of viral particles activates immune cells and triggers two distinct signal transduction pathways mediated by the adaptor proteins TRIF and MyD88. TRIF activates a signal transduction pathway responsible for the production of additional type I interferons, which are involved in antimicrobial host defense. MyD88, however, activates a signal transduction pathway responsible for the synthesis of pro-inflammatory cytokines [41]. Cytokines are small, secreted proteins involved in regulation of the immune response and communication with other immune cells. Pro-inflammatory cytokines, in particular, are primarily produced by activated macrophages and recruit leukocytes to the site of infection, enhancing the host inflammatory response [42].

However, severe infection of SARS-CoV-2 often triggers an overproduction of pro-inflammatory cytokines, deemed the “cytokine storm”. This phenomenon was previously observed in patients following the infection of SARS-CoV and MERS-CoV [43,44]. However, while elevated levels of pro-inflammatory cytokines were observed in SARS-CoV and MERS-CoV infections, patients with SARS-CoV-2 exhibited elevated levels of both pro-inflammatory and anti-inflammatory cytokines [45]. 

To date, several studies have reported increased levels of cytokines concurrent with SARS-CoV-2 infection; for example, levels of pro-inflammatory cytokines TNF-α, IFN-γ, and IL-6 and levels of anti-inflammatory cytokines IL-2, IL-4, and IL-10 were reported to be higher in COVID-19 patients than healthy individuals [46]. Reports also suggest that in certain instances, patients with severe COVID-19 were found to exhibit higher levels of pro-inflammatory cytokines such as IL-2R, IL-6, IL-8, IL-10, and TNF-α compared to patients with non-severe condition [47]. These observations indicate that upregulation of cytokines and systemic inflammation may be linked to disease severity [45,48,49,50]. A description of the main source and general function of these cytokines in the immune system is provided in Table 1.

IL-6, in particular, has been documented for its potent role in immune dysregulation in COVID-19 [51]. In the CNS, IL-6 is expressed primarily by astrocytes and microglia and is upregulated during CNS infection and neuroinflammation [52]. Moreover, prior studies have demonstrated that IL-6 may affect BBB integrity. For example, IL-6 was previously found to downregulate intraendothelial adherins and tight junction proteins in vitro, thereby increasing the paracellular permeability of human brain microvascular endothelial cells [53]. Anti-IL-6 neutralizing antibodies were also found to mitigate increases in BBB permeability due to ischemia in vivo, suggesting that IL-6 may be partially responsible for BBB dysfunction following disorders such as ischaemic injury as observed in the ovine fetus [54]. More recently, a study found that levels of IL-6 in the cerebrospinal fluid of patients with COVID-19-associated neurological symptoms were greater than that of control subjects [55]. Based on the above findings, the observed elevation of IL-6 in the cerebrospinal fluid of COVID-19 patients may be indicative of BBB dysfunction.

Along with IL-6, elevated levels of IL-8 were also found in the cerebrospinal fluid of COVID-19-associated neurological symptoms compared to control subjects [55]. In the CNS, IL-8 is expressed by endothelial cells and microglia. In this setting, IL-8 is primarily involved in leukocyte recruitment across the BBB [56]. Although the function of neutrophils in the hyper-inflammatory response of COVID-19 is yet to be established [57], it is possible that the presence of IL-8 in the cerebrospinal fluid of COVID-19 patients may provide further evidence of BBB dysfunction. Therefore, although the level of immune surveillance by neutrophils and lymphocytes in the BBB is low in the absence of infection [28], the rapid release of pro-inflammatory cytokines during the cytokine storm may trigger a heightened inflammatory response and disruption of the BBB [58]. Due to the loss of BBB integrity, the endothelial cells, pericytes, and astrocytes compromise the ability to prevent immune cells from infiltrating the brain. Rather, immune cells are able to permeate the barrier and infiltrate the CNS, possibly attacking the brain cells including the neurons. The resulting neuroinflammatory process may result in severe damage to brain function as reported [58,59].

**Table 1:** Key cytokines involved in COVID-19 cellular invasion and function in the immune system.

Cytokine	General Functions
Pro-inflammatory	
Tumor necrosis factor alpha (TNF-α)	Activation of neutrophils and platelets; enhances macrophage and natural killer cell effector function; anti-malignant cell cytotoxicity; necrosis and apoptosis [60].
Interferon gamma (IFN-γ)	Th1 cell differentiation; activation of macrophages; upregulation of class I and II major histocompatibility complex and antigen presentation; specific cytotoxic immunity; induction of antiviral enzymes; cell growth inhibition [61].
Interleukin-2 (IL-2)	T cell proliferation; long-term survival of T cells; development of T regulatory cells; NK cell growth factor; enhances NK cell cytotoxicity; antibody secretion; upregulation of B cell heavy and light chain gene expression [62].
Interleukin-6 (IL-6)	T cell growth; CD8+ T cell proliferation; differentiation of macrophages, megakaryocytes, and osteoclasts; stimulation of B cells to produce immunoglobulins [63].
Interleukin-8 (IL-8)	Recruitment and activation of neutrophils, increased expression of adhesion molecules; wound healing (stimulates migration and differentiation of fibroblasts); enhances metabolism of reactive oxygen species [64].
Anti-inflammatory	
Interleukin-4 (IL-4)	Th2 cell differentiation; immunoglobulin class switch to IgG1 and IgE; activation of alternative macrophages [65].
Interleukin-10 (IL-10)	Inhibition of pro-inflammatory cytokine production; downregulation of MHC molecules; B cell, mast cell, and thymocyte growth factor [66].

## 5. Neurological Manifestations: Case Studies

As mentioned, CNS manifestations such as ischaemic stroke, encephalitis, encephalopathy, and epileptic seizure have been observed following SARS-CoV-2 infection. Neurodegenerative diseases and inflammatory-mediated neurological disorders have also been considered as a potential risk factor of COVID-19. In this section, we present a short description and recent case studies of these neurological disorders.

### 5.1. Ischaemic Stroke

According to the Stroke Council of the American Heart Association/the American Stroke Association, ischaemic stroke is defined as, “an episode of neurological dysfunction caused by focal cerebral, spinal, or retinal infarction” [67]. Ischaemic stroke is caused by a decrease in blood flow to the brain, oxygen deprivation, and hypoxic condition. This may result from a thrombotic event, in which blood flow is disrupted due to blood vessel dysfunction, or an embolic event, in which debris blocks blood flow through the blood vessel [68].

There have been several case studies linking SARS-CoV-2 and stroke [69,70,71,72,73]. In fact, acute stroke is the most common neurological manifestation related to COVID-19 found during the neuroimaging of hospitalized patients [73]. For example, an observational study reported an elderly male who presented with disorientation, weakness of the left upper limb, and dysarthria. Prior to the development of neurological manifestations, however, the individual showed no symptoms typical of COVID-19 aside from shortness of breath. The patient had a history of chronic obstructive pulmonary disease, atrial fibrillation, and ischaemic stroke. Imaging data revealed acute anterior cerebral artery infarction. Further lab examination revealed lymphopenia, and the suspected diagnosis was severe COVID-19 pneumonia and ischaemic stroke. PCR analysis later confirmed the presence of SARS-CoV-2 [74]. 

Additionally, a retrospective study described ischaemic stroke among four patients with varying degrees of COVID-19 disease severity. All four patients had a prior history of comorbidities, namely type 2 diabetes, hypertension, or a combination. Two patients with non-severe COVID-19 presented with symptoms of stroke prior to hospitalization. The patient 1 presented with dysarthria, hemiparesis, and decreased consciousness, and the imaging data revealed sustained basilar and right superior cerebellar artery infarctions. The patient 2 presented with aphasia, hemiparesis, and hemi-sensory loss, and the imaging data indicated left middle cerebral artery infarction. The remaining two patients, initially presenting with severe COVID-19 symptoms, also developed symptoms of stroke after hospitalization, where the patient 3 presented with altered mental status and respiratory distress following an accident and COVID-19-associated acute respiratory distress syndrome. The imaging data suggested bilateral multifocal subcortical infarctions. The patient 4 presented with acute respiratory distress syndrome, septic shock, and organ failure, and the imaging data revealed right posterior cerebral artery infarction. Its noteworthy that elevated levels of D-dimer were observed commonly among all four patients [75].

Multiple factors contributing to COVID-19-associated ischaemic stroke have been proposed so far, but the exact mechanism is not yet established [76]. The most common cause of ischaemic stroke, independent of COVID-19 infection, is atherosclerosis. Atherosclerosis is an inflammatory disease that inhibits the function of endothelial cells and smooth muscle cells, resulting in vascular events [76]. However, no significant atherosclerosis disease was observed among the four patients of the retrospective study discussed above [75]. Alternatively, COVID-19 may induce a hypercoagulable state [77]. More specifically, the invasion of SARS-CoV-2 into the vascular endothelium may activate the contact and complement systems. As a result, thrombic and inflammatory cascades are activated. This may further lead to organ injury [78]. Evidence of such mechanism includes the observation of elevated D-dimer levels among all four patients described earlier in the retrospective study [75]. Moreover, recent studies have found that high levels of D-dimer were indicative of both disease severity and in-hospital mortality [79,80,81].

### 5.2. Encephalitis

Broadly defined, encephalitis refers to inflammation of the brain associated with any neurological disorder. More specifically, in research studies, encephalitis is defined as a specific form of encephalopathy (defined in Section 5.3) and at least two pieces of evidence of CNS inflammation, such as fever, seizures, CSF pleocytosis, EEG findings, or neuroimaging findings [82]. Moreover, encephalitis is classified based on etiology and timing of pathogenesis, including direct viral invasion (primary encephalitis), immune response to infection (para-infectious or post-infectious encephalitis), and unrelated to infection (autoimmune or paraneoplastic encephalitis) [83]. Several case studies of COVID-19-associated encephalitis have been documented [84,85,86], primarily involving the para-infectious and post-infectious subtype as described below.

For example, a recent case study reported an adult male who was diagnosed with para-infectious autoimmune encephalitis concurrent with severe COVID-19 pneumonia. The patient initially presented with weakness, fever, and unproductive cough. Upon hospitalization, the patient exhibited neuropsychiatric presentations, namely disorientation, aggressiveness, and stupor. The imaging data revealed signal alterations of the claustrum. SARS-CoV-2 was not detected in the patient’s cerebrospinal fluid [87]. Overall, compromised immune-inflammatory challenges in many such cases seem inevitable.

Another observational study reported an elderly female who presented with confusion and amnesia. Upon admission to the hospital, the patient had a generalized tonic-clonic seizure. Imaging data revealed T2 and fluid-attenuated inversion recovery hyper-intensities primarily in the mesial temporal lobes and medial thalimi. Subcortical white matter hyper-intensities were also observed. PCR analysis confirmed infection of SARS-CoV-2; however, the patient did not present respiratory symptoms of COVID-19. SARS-CoV-2 was not detected in the patient’s cerebrospinal fluid. These findings were indicative of limbic encephalitis. Moreover, as anti-SARS-CoV-2 IgG antibodies were negative, Zambreanu et al. suggest the observed encephalitis was para-infectious [88].

Additionally, a recent case study reported an elderly female with a prior history of Alzheimer’s disease who was diagnosed with post-infectious immune-mediated encephalitis primarily involving the brainstem. The patient initially presented with common symptoms of COVID-19, including cough, fever, and muscle pain, which improved gradually. However, the following week, the patient developed widespread involuntary movements, worsening vision, and cognitive decline. Imaging data revealed myoclonus of the limbs and tongue associated with hyperekplexia. SARS-CoV-2 was not detected in the patient’s cerebrospinal fluid [89].

The mechanism of COVID-19-associated encephalitis remains unclear; however, reports suggest that the majority of cases may be the consequence of an inflammatory-mediated mechanism [90]. In particular, cerebrospinal fluid analyses of reported COVID-19-associated encephalitis patients almost always revealed an absence of SARS-CoV-2 [91,92], as discussed above [87,88,89]. Because SARS-CoV-2 is very rare in the cerebrospinal fluid, direct viral invasion of the CNS is an unlikely mechanism, although not impossible [92,93]. The presentation of limbic encephalitis discussed in the second observational study also supports the hypothesis of an inflammatory-mediated mechanism [88,92]. SARS-CoV-2 may act as a viral trigger, in which the pro-inflammatory state following infection “primes” the immune cells of CNS for over-reactivity. The result is an autoimmune response against the host CNS [94].

The cytokine profile of patients with COVID-19-associated encephalitis exhibit further evidence of neuroinflammatory sequelae. Pilotto et al. measured cytokine levels in the serum and cerebrospinal fluid of a patient with COVID-19-associated meningoencephalitis. Prior to the administration of steroid therapy, levels of IL-8 and IL-6 were higher in the cerebrospinal fluid than the serum. Glial and astrocytic markers, namely TREM-2, YKL-40, and CNS-specific GFAP were also elevated. The authors suggest that these laboratory findings are indicative of intrathecal mode of activate inflammation [90,95]. Together, these findings suggest that SARS-CoV-2 infection and subsequent inflammatory response could trigger BBB dysfunction, leading to immune cell infiltration and CNS tissue damage. Prior studies have also implicated compromised BBB integrity in tick-borne encephalitis, Japanese encephalitis [96,97].

Finally, the third case study discussed above brings attention to the potential for post-infectious immune-mediated neurological sequelae. Although it is challenging to prove SARS-CoV-2 was the cause of encephalitis, Khoo et al. suggest that infection may have contributed to disease pathogenesis as the patient developed severe symptoms one week after COVID-19 [89]. Moreover, observed post-infectious immune-mediated neurological sequelae have not been limited to encephalitis. For example, recent studies have reported cases of acute disseminated encephalomyelitis [98,99,100] and multiple sclerosis [101] following SARS-CoV-2 infection. Direct causation has not been established; however, these observations demonstrate that neuroinflammation following SARS-CoV-2 may have severe long-term consequences, many of which still remain unknown.

### 5.3. Other Encephalopathies

Encephalopathy refers to an altered mental status caused by pathology that affects brain function. This altered mental status may include disorientation, short-term memory loss, inattentiveness, and/or abnormal arousal [102]. More specifically, in research studies, encephalopathy is defined as altered consciousness lasting over 24 h, including personality and behavioral changes, lethargy, and irritability [82]. There have been several cases of COVID-19-associated encephalopathy documented [55,103,104,105].

For example, an observational study described an elderly male with a prior history of coronary artery disease, hypertension, and type II diabetes who initially presented with cough and fatigue. PCR analysis confirmed infection of SARS-CoV-2, and chest imaging indicated COVID-19 pneumonia. Days after hospitalization, the patient became unresponsive to commands, and stimuli-induced motor responses in the extremities were absent. The imaging data revealed multi-compartmental hemorrhages and mild surrounding vasogenic edema. After further examination, the patient was diagnosed with para-infectious hemorrhagic encephalopathy. SARS-CoV-2 was not detected in the patient’s cerebrospinal fluid [106].

Additionally, a recent case series reported that various features of encephalopathy were the most common neurological symptom observed among six hospitalized patients with confirmed COVID-19 and neurological abnormalities. In this case series, one patient presented with disorientation and poor spatial awareness, in which the imaging data revealed evidence of small-vessel disease and global cortical atrophy. Another patient exhibited disorientation regarding time and place, poor memory, speech difficulties, fatigue, and seizures. The imaging data revealed atherosclerotic changes indicative of the patient’s comorbidities. Furthermore, the third patient demonstrated altered personality, cognitive slowing, and verbal difficulties, in which the imaging data revealed white matter changes. Lastly, one among the six subjects presented with symptoms such as disorientation regarding time, person, and situation, and significant verbal difficulties [93].

According to recent literature, it is currently under debate whether COVID-19-associated encephalopathy is due to a direct consequence of SARS-CoV-2, a pro-inflammatory-thrombic state, or a combination of the two [107]. However, as observed with encephalitis, evidence suggests that an inflammatory-mediated mechanism may contribute to disease pathogenesis. For example, in the first observational study, a cerebrospinal fluid cytokine assay revealed elevated levels of the soluble receptors for IL-1, IL-6, soluble glycoprotein-130, and TNF-α, indicating pro-inflammatory cytokine dysregulation. Based on this finding, Krett et al. hypothesize that para-infectious inflammation may contribute to hemorrhaging encephalopathy [106]. Additionally, the second case-series reported that neopterin and β_2_M, two indicators of immune activation, were elevated in the cerebrospinal fluid samples of patients with COVID-19 and neurological abnormalities [93].

Moreover, Farhadian et al. reported elevated levels of IL-6, IL-8, and interferon-gamma induced protein in the cerebrospinal fluid and serum of an elderly woman with COVID-19-associated encephalopathy. A higher level of Monocyte Chemoattractant Protein-1 was also found in the patient’s cerebrospinal fluid compared to control subjects. More specifically, Monocyte Chemoattractant Protein-1 is a pro-inflammatory chemokine expressed by neurons, astrocytes, and microglia that is involved in recruiting “inflammatory infiltrate” into the CNS [108]. Therefore, this finding suggests potential immune cell infiltration into the brain; however, it remains unclear whether BBB dysfunction contributes to such disease pathogenesis. This is because the albumin ratio reflecting BBB integrity was normal among all six patients described in the second case series above, indicating an absence of BBB disruption [93] or possibly a minimal BBB leakage in such a case. Therefore, further research is necessary to elucidate the mechanism of COVID-19-associated encephalopathy.

### 5.4. Epilepsy

Epilepsy is a condition characterized by unprovoked and repetitive seizures resulting from the imbalance of excitatory and inhibitory neuronal activity [109,110,111]. Seizures concurrent with SARS-CoV-2 infection have been reported, although not extensively [112,113,114]. For example, a recent observational study described a young adult who initially presented with myalgia, lethargy, fever. The patient was admitted to the hospital following a generalized tonic-clonic seizure, and cerebrospinal fluid analysis revealed lymphocytic pleocystosis. Further imaging was unremarkable except for sphenoid sinus, in which mild mucosal thickening was observed. Although PCR analysis initially denied the presence of SARS-CoV-2, a second examination confirmed the patient’s infection. However, it was noted that the patient lacked respiratory symptoms associated with COVID-19, the overall clinical course was mild, and SARS-CoV-2 was not detected in the patient’s cerebrospinal fluid [115].

Additionally, another recent study reported an adult male who presented with bulging of the scalp flap, loss of smell, and numbness of the left hand and face. The patient had a prior history of symptomatic epilepsy, but was seizure-free for five months on a medication regimen. Upon admission to the emergency room, the patient had a focal aware seizure originating in his left hand and fever. Imaging data revealed swelling of the right temporal lobe, although no cerebral infarction nor new vessel obstruction was observed. PCR analysis confirmed infection of SARS-CoV-2. Kadono et al. suspected the presence of severe brain edema was likely due to the neuroinvasion of SARS-CoV-2 or a cerebrovascular event resulting from a hypercoagulable state [116].

Moreover, a retrospective case study conducted from early-April to mid-May 2020 found an incidence of seizure among 0.7% of 1043 COVID-19 patients. Of the seven patients observed, four patients presented with new-onset seizures; the remaining had a history of well-controlled epilepsy. However, three patients lacked COVID-19 symptoms before the occurrence of seizure. Imaging data of an adult female with status epilepticus revealed gyriform diffusion restriction and extensive leukoencephalopathy most likely due to the patient’s condition or viral encephalitis. Evidence of SARS-CoV-2 infection and inflammation was not detected in the patient’s cerebrospinal fluid [117].

There are several potential mechanisms by which SARS-CoV-2 infection may cause seizures. These could manifest due to cerebral damage from SARS-CoV-2 neuroinvasion, hypoxia, metabolic derangements, organ failure, or therapeutic interventions [118]. As discussed, the patient in the first observational study was diagnosed with lymphocytic pleocytosis [115]. This finding suggests that BBB dysfunction following SARS-CoV-2 infection and inflammatory response may cause abnormal neuronal activity due to an imbalance of ions, transmitters, and metabolic products, thereby resulting in seizure [119]. Moreover, previous studies have concluded that compromised BBB integrity induced seizures in both human and animal subjects [120,121,122]. It must be noted, however, that no evidence of inflammation was detected in the cerebrospinal fluid samples of the patient described in the retrospective study above [117]. Additional research is required to explain these results and fully understand the mechanism of COVID-19-associated seizures.

### 5.5. Neurodegenerative Disease and Inflammatory-Mediated Neurological Disorder

There is limited evidence on COVID-19-induced neurodegenerative disease reported thus far [101,123]. Some neurodegenerative diseases and inflammatory-mediated neurological disorders, including multiple sclerosis (MS), Parkinson’s disease, and Alzheimer’s disease, have been investigated as potential risk factors of COVID-19. However, it is important to note that the findings discussed below are preliminary hypothesis; no consistent findings support the association between neurodegenerative disorders and COVID-19.

#### 5.5.1. Multiple Sclerosis

MS is an autoimmune disease involving inflammation, demyelination, gliosis, and neuronal loss of the CNS [124]. It is well-established that patients with MS have an increased risk of infection, which may cause morbidity or neurological symptoms linked to BBB dysfunction. Patients receiving second generation disease-modifying therapies may be particularly susceptible to infection due to the immunosuppressive and immunomodulatory effects of the drugs [125,126]. On the other hand, several first-line treatments of MS, including interferons, are considered very low-risk and may instead aid in immunity [127].

Studies investigating MS as a potential risk factor of COVID-19 have reported conflicting findings. For example, a small cohort study conducted in Veneto, Italy found the prevalence of COVID-19 was approximately 2.5 times greater among individuals with MS (1%) compared to the general population (0.4%) [128]. However, a large-scale survey found that regardless of a disease-modifying drug regimen, patients with MS did not exhibit an increased risk of COVID-19 [129]. Additionally, a cohort study revealed no association between exposure to disease-modifying therapies and COVID-19 disease severity among MS patients [130]. It must be considered, however, that the latter findings may be due to preventative measures adopted to protect this particularly vulnerable group [129]. Moreover, the former study was conducted on a small sample size, limiting the scope of its findings [128]. As demonstrated by these conflicting findings, it is currently under debate whether MS is a risk factor of COVID-19 [131].

Furthermore, prior studies have shown viral infection may result in MS exacerbations and relapses. In particular, infection may act as an environmental risk factor, triggering autoimmunity and MS among genetically predisposed individuals [132,133,134]. For example, optic neuritis was observed in a young adult following SARS-CoV-2 infection. However, Palao et al. hypothesize that MS disease pathogenesis was ongoing at the time of infection, and SARS-CoV-2 served only as a precipitating factor [101]. Evidently, further research is required to understand the complex relationship between MS and COVID-19.

#### 5.5.2. Parkinson’s Disease

Parkinson’s disease is neurodegenerative disorder of the basal ganglia, primarily resulting in bradykinesia, resting tremor, or rigidity [135]. A recent cohort survey found that COVID-19 was prevalent among 0.9% of a population of individuals with Parkinson’s disease. This percentage is greater than Tuscany’s regional frequency of 0.25%, the location of the study and multi-centered survey conducted [136]. Additionally, another cohort study reported a higher mortality rate from COVID-19 among patients with Parkinson’s disease (19.7%, mean age 71.4) compared to the general population of individuals over 70 (12.8%) [137]. However, a large-scale telephone survey conducted in Lombardy, Italy revealed that the prevalence of COVID-19 among patients with mild to moderate Parkinson’s disease (7.1%) was similar to the prevalence of COVID-19 among the general population (7.9%). There was no significant difference in morbidity or mortality between the two populations [138].

There is currently no clear evidence that Parkinson’s disease itself increases the risk of COVID-19. This is because patients with Parkinson’s disease are often susceptible to age-related comorbidities, such as hypertension, diabetes, coronary artery disease, and cerebrovascular disease, which may predispose the individual to more severe infection [136,139]. Moreover, the elderly exhibit higher levels of pro-inflammatory cytokines, namely TNF-α, IL-1, and IL-6 [140]. As discussed in the section “Cytokine Storm on the Blood-brain barrier: a Malfunction of the Immune System”, these cytokines are key players in the heightened inflammatory response associated with severe condition and neurological damage. Therefore, additional research is necessary to reveal whether the observed increase in COVID-19 prevalence and mortality among individuals with Parkinson’s disease is a consequence of the neurodegenerative disease itself or other confounding factors, such as comorbidities and increased age.

#### 5.5.3. Alzheimer’s Disease

Alzheimer’s disease is another debilitating neurodegenerative disease resulting from neuronal cell death. Symptoms of Alzheimer’s include behavioral and cognitive impairment, including but not limited to loss of memory, judgement, and comprehension [141]. A retrospective study reported that among a cohort of COVID-19 patients with pneumonia, the mortality rate among patients with dementia (62.2%) was greater than that of patients without dementia (26.2%) [142].

It is currently unknown whether Alzheimer’s disease is a risk factor of COVID-19; however, Naughton et al. hypothesize that SARS-CoV-2 may predispose asymptomatic or pre-symptomatic individuals to neurodegeneration as a consequence of silent viral entry or systemic inflammation [140]. In particular, a prior study demonstrated that amyloid fibrils, which induce interferon expression, contained nucleic acids and were likely involved in the innate immune response. Patients with Alzheimer’s disease were also found to exhibit increased levels of interferon-induced genes. From these findings, it is proposed that amyloid fibrils may capture pathogens, such as SARS-CoV-2, activate microglia, and thereby trigger a pro-inflammatory interferon response [140,143]. However, based on literature, till date the potential relationship between Alzheimer’s disease and COVID-19 remains unclear.

## 6. Conclusions and Future Directions

Although SARS-CoV-2 primarily attacks the respiratory tract, cases of COVID-19-associated neurological disorders are a possibility, especially among patients with severe disease progression. Although the exact mechanisms of disease pathogenesis remain unknown, studies have often implicated potential contribution of neuroinvasion, neuroinflammation, and BBB dysfunction in the development of these CNS manifestations. Certain neurodegenerative diseases and inflammatory-mediated neurological disorders may also serve as a risk factor for COVID-19; however, the direct link and long-term consequences have yet to be established and conditions with disease comorbidities could increase the susceptibility of an individual to such infection. Many studies conducted have been case reports, with a few significant clinical trials. Therefore, further research is necessary to elucidate the detail mechanism of SARS-CoV-2 infection leading to neurological disorders. More specifically, if SARS-CoV-2 truly utilizes a combination of pathways for CNS invasion, it may be interesting to explore whether certain pathways are linked to the manifestation of certain neurological disorders, which is yet to be determined. Future studies will also unveil other potential mechanisms for viral invasion, such as the lymphatic system and gastrointestinal-CNS linkage.

Moreover, a recent study has suggested an alternative hypothesis to the cytokine storm: a renin angiotensin mediated bradykinin storm. Garvin et al. proposed that SARS-CoV-2 may downregulate angiotensin-converting enzyme and upregulate ACE2. Due to decreased degradation of bradykinin by angiotensin-converting enzyme, bradykinin levels increase, resulting in vascular permeability, hypokalemia, and acid buildup in the lungs. The bradykinin storm also provides explanations to many of the neurological symptoms of COVID-19, such as encephalopathy, dizziness, headache, cognitive impairment, and ischemia [144]. Therefore, further investigation into the bradykinin storm may provide information which would be beneficial to both scientific understanding and therapy development.

Furthermore, although neurological manifestations are often associated with severe disease progression, recent reports have demonstrated that CNS disorders may develop among certain patients with mild to moderate COVID-19 clinical courses. These manifestations may even present themselves prior to respiratory symptoms of COVID-19, as documented in a few case studies discussed. Therefore, while it is essential for health care professionals to consider neurological disorders as a possibility among COVID-19 patients with severe illness, the medical community must not overlook the possibility of neurological damage among individuals with mild to moderate infection.
